# A systematic review of suture-button versus syndesmotic screw in the treatment of distal tibiofibular syndesmosis injury

**DOI:** 10.1186/s12891-017-1645-7

**Published:** 2017-07-04

**Authors:** Pei Zhang, Yuan Liang, Jinshan He, Yongchao Fang, Pengtao Chen, Jingcheng Wang

**Affiliations:** 1 Dalian Medical University, Dalian, Liaoning 116044 China; 2 Department of Orthopedics, Clinical Medical College of Yangzhou University, Subei People’s Hospital of Jiangsu Province, Nantong West Road 98, Yangzhou, 225001 China

**Keywords:** Syndesmosis, Ankle, Suture-button, TightRope, Screw

## Abstract

**Background:**

Now, using a suture-button device to treat distal tibiofibular syndesmotic injuries is overwhelming due to its advantages over screw fixation. Current systematic review was conducted to make a comparison between suture-button fixation and traditionally screw fixation in the treatment of syndesmotic injuries. The outcomes included functional outcomes, implant removal, implant failure, malreduction, post-operative complications (except implant failure and malreduction), and cost-effectiveness aspects.

**Method:**

A literature search in the electronic databases of Medline, Embase, the Cochrane Library, Web of Science was conducted to identify studies until March 2017. The references of the included articles were also checked for potentially relevant studies. Only English articles were included. We followed the Preferred Reporting Items for Systematics reviews and Meta-Analysis (PRISMA) guidelines in this review.

**Results:**

Finally, 10 studies were identified, encompassing a total of 390 patients. The mean American Orthopaedic Foot and Ankle Society ankle score (AOFAS) score of 150 patients treated with the suture-button device was 91.06 points, with an average follow-up of 17.58 months, and the mean AOFAS score of 150 patients treated with syndesmotic screws was 87.78 points, with an average follow-up of 17.73 months. Implant removal was reported in 5 of 134 (3.7%) patients treated with the suture-button device, and in 54 of 134 (40.2%) patients treated with the syndesmotic screw. No patient in the suture-button fixation group had implant failure, however the rate of implant failure in the screw fixation group was 30.9%. Malreduction was reported in 1 of 93 (1.0%) patients treated with the suture-button device, and in 12 of 95 (12.6%) patients treated with the syndesmotic screw. The rate of post-operative complications in the suture-button fixation group was 12.0% and 16.4% in the screw fixation group. There was only one publication demonstrated about cost-effectiveness aspects, it showed that patients treated with the suture-button device spent on average $1482 less and had a higher quality of life by 0.058 quality-adjusted life-year compared with patients who received fixation with 2 syndesmotic screws in supination-external rotation type 4 injuries.

**Conclusion:**

Based on our research, though the suture-button fixation group had similar functional outcome (measured on the AOFAS score) and post-operative complication rate compared with the syndesmotic screw fixation group, the suture-button device could lead to better objective range of motion (ROM) measurements and earlier return to work. Besides, the suture-button fixation group had lower rate of implant removal, implant failure, and malreduction. However, high-quality randomized controlled trials with more uniformity in outcome reporting are desirable to determine the long-term effects and cost-effectiveness of the suture-button device.

## Background

Syndesmotic injuries arise in approximately 13% of all patients with ankle fractures which are commonly seen in pronation and external rotation injuries, and in approximately 20% of ankle fractures requiring operative fixation [1]. As persistent ankle pain, function disability, and early osteoarthritis are potential problems related to misdiagnosed or inadequate treatment of syndesmotic injuries [2, 3], thus, it is essential to acquire accuracy and maintenance of syndesmotic reduction when treating ankle fractures with concomitant syndesmotic injuries.

Though screw fixation as the gold-standard in treatment of syndesmotic injury, some significant issues should be considered, such as screw loosening, breakage, discomfort, reoperation, loss of reduction due to early implant removal [4–7]. More recently, the suture-button fixation device has aroused the attention of many orthopedists, especially TightRope. This device has been reported with some potential advantages, such as allowing of physiological movement while retaining the required reduction, less risk of implant removal and recurrent syndesmotic diastasis, and earlier rehabilitation [8–10]. Anatomic reduction has been shown to be the most important predictor of clinical outcomes [11]. Optimal surgical management is still a subject of debate in the literature [2, 12]. Therefore, current systematic review was undertaken to make a comparison between suture-button fixation and screw fixation focusing on following outcomes: functional outcome, need for implant removal, implant failure, rate of malreduction, post-operative complications rate (except implant failure and malreduction), and cost-effectiveness aspects.

## Methods

The study was conducted according to the Preferred Reporting Items for Systematic Reviews and Meta-Analyses criteria (PRISMA).

### Eligibility criteria

To be included in our analysis, the study had to: (1) evaluate a comparison between suture-button fixation and traditionally screw fixation in the treatment of syndesmotic injuries. (2) the studies included at least one of the outcome measures. Studies were excluded: (1) case reports, conference abstracts, and publications that were only discussing screw fixation or suture-button. (2) pediatric or cadaveric studies (3) studies not published in English. (4) duplicate publication.

### Information sources and search

A literature search was conducted to identify studies in which made a comparison between suture-button fixation and traditionally screw fixation in the treatment of distal tibiofibular syndesmotic injuries. The following key terms were combined with Boolean operators in the search: “syndesmo*”, “tibiofibular”, “TightRope”, “suture button”, “screw”, The last search was updated on March 2017. We only included English articles. An additional search of the references of included studies was performed to find relevant studies.

### Data collection

The following data were independently extracted from each of the included studies by two investigators (Y Liang and YC Fang): name of the first author, year of publication, targeted population, study type, mean-age, suture-button usage, cortical screw usage, rehabilitation process, follow-up, and the outcomes of the systematic review including: main functional outcomes, implant removal, implant failure, malreduction, post-operative complications (except implant failure and malreduction), and key findings. When disagreement existed, it was resolved by consulting another investigator (PT Chen).

### Quality assessment

The quality of the randomized controlled trials (RCTs) was assessed according to the Cochrane risk assessment scale, including details of the methods of random sequence generation, allocation concealment, blinding, incomplete outcome data, selective outcome reporting, and other sources of bias. The Methodological Index for Non-Randomized Studies (MINORS) Criteria was used to assess non-RCTs and was scored from 0 to 24 [13]. The assessments were performed by two investigators independently (Y Liang and YC Fang). Any disagreement was resolved by a third reviewer (JS He).

## Results

### Study selection

**Fig. 1:**
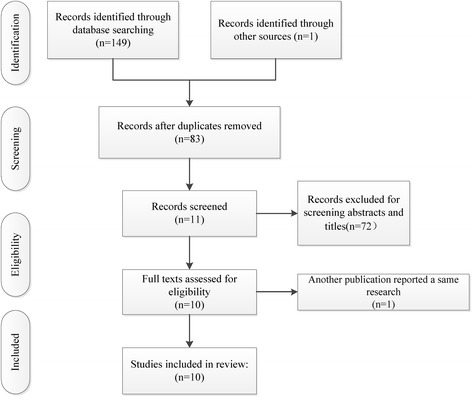
The flow chart of studies selecting

A total of 150 potentially relevant references preliminarily reviewed. By scanning the titles and abstracts, 11 articles that met the inclusion criteria were reviewed for full-text screening. After full texts assessed for eligibility, 1 article [14] was excluded, because the main topic of it was the introduction of TightRope fixation technique, besides, another publication [15] demonstrated a same research. Finally, a total number of 10 eligible articles [2, 4, 8, 9, 11, 12, 15–18] that are described in this systematic review. The suture-button fixation group included 196 participates, and 194 patients in the screw fixation group. The selection process was shown in Fig. 1.

### Quality of the included studies

**Fig. 2:**
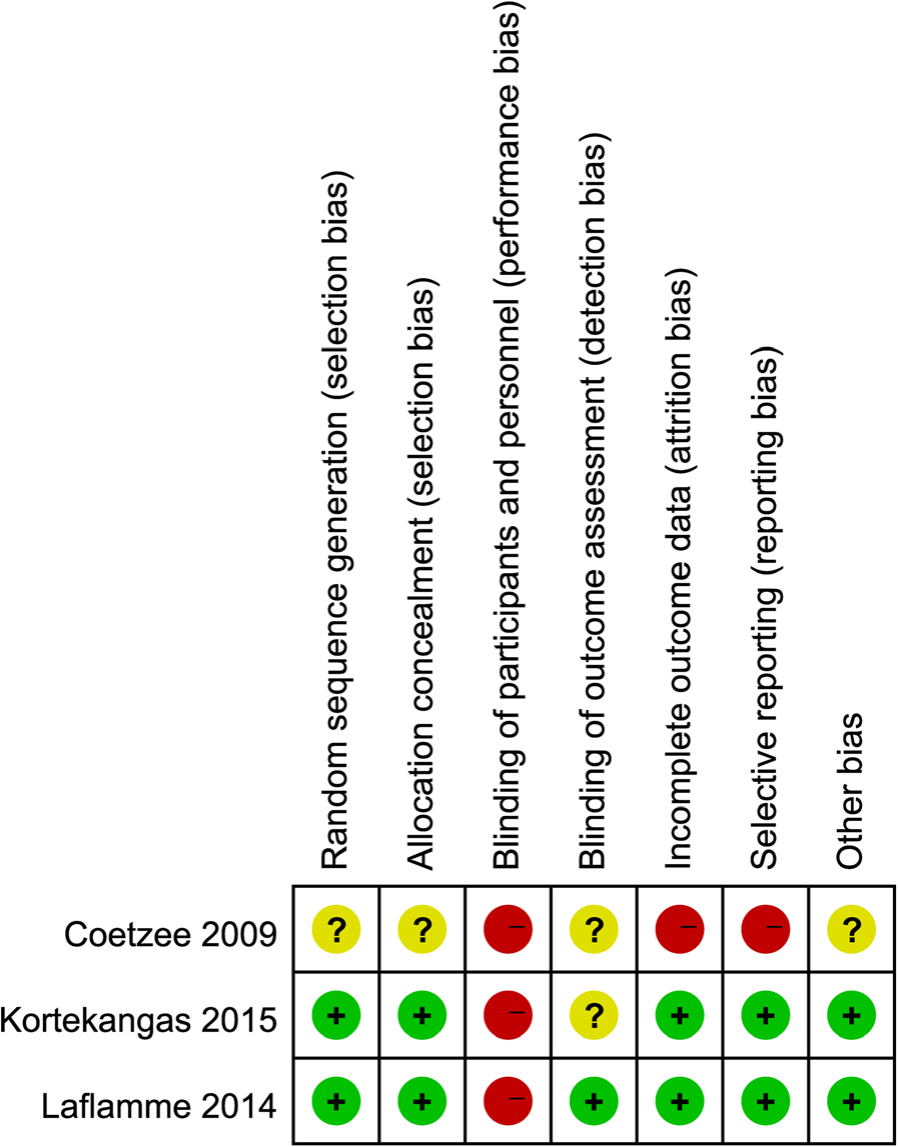
The quality of the randomized controlled trials

Each included randomized controlled trials [8, 9, 15] showed clear inclusion and exclusion criteria. In two [8, 9] of the included RCTs, the randomization algorithm was generated from a computer. The allocation concealment was performed using opaque sealed envelopes in two RCTs [8, 9]. None of them provided the information of double blinding. One Publication [9] provided binding of outcome assessment. All RCTs except one study [15] reported complete outcome data. Intent-to-treatment analysis was performed in two RCTs [8, 9], thus, a potential risk for typeIIstatistical error would exist. The publication concerning cost-effectiveness was of Level II evidence. The results of the quality of the included RCTs were shown in Fig. 2. The studies had an average MINORS score of 16.33 ± 2.05, which indicated a fair quality of evidence. The detailed results of MINORS scoring were presented in Table 1.

**Table 1:** The characteristics of the included studies (a)

Study (year)	Targeted population	Study type	Number: SBG VS SG	Mean age (years): SBG VS SG	Suture-button usage	Cortical screw usage	Follow-up	MINORS score
Kocadal 2016 [4]	Turkey Ankara,	Retrospective comparative study	26/26	43.3/44.8	1 ZipTight Fixation System	One 3.5mm screw (4 cortices)	16.7 ± 11.0 months	18
Kim 2016 [2]	Korea Busan	Historical control study	24/20	51.3/40.5	1 TightRope implant	One 3.5mm screw (3 cortices)	13.4 / 14.6 months	12
Seyhan 2015 [17]	Turkey Istanbul	Retrospective comparative study	15/17	33.2 /32.0	1 TightRope implant	One 4.5 mm screw (4 cortices)	14.6 (12–50) months	17
Kortekangas 2015 [8]	Finland Oulu	Prospective randomized controlled clinical trial	21/19	46.0 /43.5	1 TightRope implant	One 3.5 mm screw (3 cortices)	At least 2 years, mean 36 months in TightRope group,37 months in the syndesmotic screw group)	—
Laflamme 2015 [9]	Netherland And Canada.	Prospective Randomized Multicenter Trial	34/36	40.1/ 39.3	1 TightRope implant	One 3.5mm screw (4 cortices)	12 months	—
Naqvi 2012 [11, 25]	Ireland Drogheda,	Cohort study	23/23	42/40	16 cases with one TightRope implant 7 cases with two TightRope implant	20 cases with one screw 3 cases with two screws (4 cortices)	2.5 years	16
Cottom 2009 [12]	America Columbus	Prospective cohort study	25/25	34.68/36.68	21 cases with a single interosseous suture endobutton 4 cases with dual interosseous suture endobuttons	12 cases with a single screw 13 cases with 2 screws	10.78 months /8.2 months	17
Coetzee 2009 [15]	America Minneapolis	Prospective, randomized clinical trial	12/12	35/38	All but one had two TightRopes	4.0 mm, 4.5 mm and 6.5 mm screws	2.3year	—
Thornes 2005 [18]	Ireland	Retrospective cohort study	16/16	32/31	A suture-button (One #5 braided polyester suture and two endobuttons)	One four-cortical syndesmosis screw	12 months	18

*SBG* suture-button group, *SG* screw group

### The characteristics of the included studies

The main characteristics of 9 studies were shown in Tables 1, 2 and 3. Below is a summarized description for the 9 included articles.

**Table 2:** The characteristics of the included studies (b)

Study (year)	Main functional evaluation	Mean scores SBG VS SG	Implant removal SBG VS SG	Implant failure SBG VS SG	Malreduction SBG VS SG	Complications SBG VS SG	Routine screw removal (yes or no)
Kocadal et al. 2016 [4]	AOFAS	88.4 /86.1	1/10	0/1	NR	2 (1 low-grade infection and implant irritation)/1 reflex sympathetic dystrophy	No
Kim et al. 2016 [2]	AOFAS	88.1/86.6	NR	0/5	NR	NR	NR
Seyhan 2015 [17]	AOFAS	93.73/93.35	2/17	0/0	0/0	6 (2 Implant discomfort and 4 soft tissue irritation )/2 Implant discomfort	Yes
Kortekangas 2015 [8]	Olerud–Molander score	82/84	1/3	0/16(broken in three patients and loosened in 13 patients)	1/3	1 post-operative infection/3 local irritation	No
Laflamme 2015 [9]	Olerud–Molander score	93.3/ 87.7	2/11	0/13	0/4	3(two superficial infection and one partial syndesmosis ossification)/12(1 partial syndesmosis ossification and 11 discomfort)	No
Naqvi 2012 [11, 25]	AOFAS	89.56/86.52	NR	NR	0/5	NR	Yes
Cottom 2009 [12]	Modified AOFAS (a maximum of 63 possible points)	50.64/53.45	0/17	0/12 (screw loosening in 5 patients and 7 cases of screw breakage)	NR	NR	No
Coetzee 2009 [15]	AOFAS	94/88	1/1	0/1	NR	1 superficial infection/0	No
Thornes 2005 [18]	AOFAS	93/ 83	0/12	NR	NR	No major complications or wound infections	No

*AOFAS* American Orthopaedic Foot and Ankle Society ankle score, *SBG* suture-button group, *SG* screw group, *NR* no report

**Table 3:** The characteristics of the included studies (c)

Study (year)	Rehabilitation process (SBG)	Rehabilitation process (SG)	Time to full weight bearing (weeks) SBG VS SG	Key findings
Kocadal et al. 2016 [4]	Short leg splints for 3 weeks, after splint removal, partial weight bearing was allowed. At the sixth postoperative week, full weight bearing was allowed	Short leg splints for 3 weeks, after splint removal, partial weight bearing was allowed. At the sixth postoperative week, full weight bearing was allowed	NR	Although the functional outcomes were similar, the restoration of the fibular rotation in the treatment of syndesmotic injuries by screw fixation was troublesome and the volume of the distal tibiofibular space increased with the suture-button fixation technique.
Kim et al. 2016 [2]	A below-the-knee cast for 1 week, partial weightbearing at 6weeks postoperatively	A below-the-knee cast for 1 week, partial weightbearing 6 to 8 weeks postoperatively	NR	Both suture-button and metal screw fixation are effective treatment methods for an ankle fracture accompanied by syndesmotic injury.
Seyhan 2015 [17]	Plaster-splint for two Weeks and then pressure-socks for 4 weeks Partial weight bearing using double crutches and then complete weight bearing at the end of the 3rd month	Plaster-splint for two Weeks and then pressure-socks for 4 weeks Partial weight bearing using double crutches and then complete weight bearing at the end of the 3rd month (after screw removal)	NR	Elastic fixation is as functional as screw fixation in the treatment of ankle syndesmosis injuries. The unnecessary need of a second surgical intervention for removal of the fixation material is another advantageous aspect of this method of fixation.
Kortekangas 2015 [8]	A below-the-knee cast with the ankle joint at a 90° for 6 weeks with partial weight bearing. At 6 weeks, the cast was removed, the ankle was examined, and a research physiotherapist instructed the patient in rehabilitation exercises. No additional bracing was used and weight bearing was allowed as tolerated	A below-the-knee cast with the ankle joint at a 90° for 6 weeks with partial weight bearing. At 6 weeks, the cast was removed, the ankle was examined, and a research physiotherapist instructed the patient in rehabilitation exercises. No additional bracing was used and weight bearing was allowed as tolerated	NR	Syndesmotic screw and TightRope had similar postoperative malreduction rates. After at least 2 years of follow-up, malreduction rates may slightly increase when using trans-syndesmotic screw fixation, but reduction was well maintained when fixed with TightRope. Neither the incidence of ankle joint osteoarthritis nor functional outcome significantly differed between the fixation methods.
Laflamme 2015 [9]	No weight bearing in a cast for 6 weeks and then rehabilitation without protection	No weight bearing in a cast for 6 weeks and then rehabilitation without protection	NR	Dynamic fixation seems to result in better clinical and radiographic outcomes. The implant offers adequate syndesmotic stabilization without failure or loss of reduction, and the reoperation rate is significantly lower than with conventional screw fixation.
Naqvi 2012 [11, 25]	All patients were immobilized in a below-the-knee, nonweightbearing cast for 6 weeks, followed by physical therapy and weightbearing as tolerated	All patients were immobilized in a below-the knee, nonweightbearing cast for 6 weeks, followed by physical therapy and weightbearing as tolerated	8.0/9.1	TightRope provides a more accurate method of syndesmotic stabilization. Syndesmotic malreduction is the most important independent predictor of clinical outcomes.
Cottom 2009 [12]	A non–weight-bearing splint for 10 days postoperatively, and a weight-bearing cast was maintained for 3 additional weeks until transfer into a removable boot walker with full weight bearing to tolerance	A non–weight-bearing splint for 10 days postoperatively, and a weight-bearing cast was maintained for 3 additional weeks until transfer into a removable boot walker with full weight bearing to tolerance	5.52/10.52 4.93/9.5 (the Maisonneuve fracture group and the isolated soft tissue ligamentous injuries were analyzed separately)	Interosseous suture with endobuttons is a reasonable option for repair of ankle syndesmotic injuries, and may be as effective as traditional internal screw fixation
Coetzee 2009 [15]	A short leg cast splint for two weeks with nonweightbearing and then a pneumatic Cam boot was applied for partial weightbearing . At six weeks, Cam boot removal for weightbearing if the syndesmosis appears stable and any associated fractures were healed	A short leg cast splint for two weeks with nonweightbearing and then a pneumatic Cam boot was applied for partial weightbearing . At six weeks, Cam boot removal for weightbearing if the syndesmosis appears stable and any associated fractures were healed	NR	The TightRope® fiber wire fixation group had a statistically significant better range of motion compared to conventional screw fixation. The AOFAS ankle and hindfoot score did not show a significant difference between the two groups at medium term follow-up.
Thornes 2005 [18]	A below-knee cast for 6 weeks and then full weightbearing at 6 weeks postoperatively after cast removal. (2 weeks, patients with stable plate osteosynthesis of the fibula fracture were allowed partial weightbearing up to 50% of body weight with a below-knee cast)	A below-knee cast at least for 6 weeks and then full weightbearing at 6 weeks postoperatively after cast removal.	NR	Suture-button fixation is simple, safe, and effective. Patients have had improved outcomes and faster rehabilitation, without needing routine implant removal.

*SBG* suture-button group, *SG* screw group, *NR* no report

Kortekangas et al. [8] published a prospective randomized controlled trial comparing TightRope fixation (*n* = 21) with screw fixation (*n* = 19), predominantly Weber C fractures. A standing cone-beam CT-scan with bone algorithm was performed at final follow-up to qualify the grade of osteoarthritis. No significant differences in functional results between groups were detected at the last follow-up. However, all functional scores were lower in patients who had malreduced syndesmosis on final follow-up than in patients with anatomically reduced syndesmosis. CT evaluation showed a low malreduction rate in both groups and both methods maintained reduction well. The incidence of osteoarthritis showed no significant difference between the two groups.

Laflamme et al. [9] conducted a prospective randomized multicenter trial, comparing the clinical and radiographic outcomes after reparation of an acute syndesmosis rupture with either a 3.5-mm cortical screw (36 patients) or a TightRope (34 patients). The fracture types were 44-B2, 44-B3, 44-C1 and 44-C2. Regarding the clinical outcomes: (1). both groups had good to excellent Olerud–Molander scores (80 or higher) at 12 months, but the increase was faster and higher in TightRope fixation group. The AOFAS score showed the similar result; (2). The TightRope fixation group had no significantly better plantar flexion (*p* = 0.45) and dorsal flexion (*p* = 0.43) at last follow-up, however, the ankle range of motion was higher in the dynamic fixation group at all times when considering the plantar flexion, although this difference was minor for dorsal flexion.; (3). Patients in the TightRope fixation group seemed to be with less pain according to visual analogue scale (VAS); Regarding radiologic results, though adequate reduction was achieved after the surgery in both groups except for 1 patient in the screw group who required a corrective surgery (screw removal and new screw positioning, with good final reduction), patients in the screw fixation group had significantly higher loss of reduction (*P* = 0.0005). Significant loss of reduction (the lateral tibiofibular clear space more than 6.0 mm) was observed in 4 patients (3 cases occurred after screw removal) in the static fixation group. However, the TightRope fixation group showed no significant loss of reduction. The reoperation rate was significantly higher in the static fixation group (*P* = 0.006) Both groups had 1 patient with partial syndesmotic ossification.

Naqvi et al. [11] published a cohort study comparing the accuracy and maintenance of syndesmotic screws versus the TightRope system. Twenty-three patients were included in each group, predominantly with Weber C injuries. There was no significant difference between the tightrope and syndesmotic screw groups in mean postoperative AOFAS score or Foot and Ankle Disability Index (FADI) score. CT-scan evaluation demonstrated a significant 21.7% higher risk of malreduction in the screw fixation group. The average time to full weight-bearing was 8 weeks in the TightRope group and 9.1 weeks in the syndesmotic screw group. They made a regression analysis confirmed that malreduction of syndesmosis as the only independent variable that affected the clinical outcome (regression coefficient, −12.39; *t* = −2.43; *P* = 0.02).

Kocadal et al. [4] performed a retrospective comparative study, including 27 lateral malleolar fractures, 20 bimalleolar fractures and 5 trimalleolar fractures with syndesmotic injury, of which 26 patients were treated with the TightRope system and 26 patients with the cortical screw system. Radiologic evaluations were performed by postoperative CT scans. There was no statistically significant difference in the functional ankle joint scores between the groups. They pointed out that the restoration of the fibular rotation in the treatment of syndesmotic injuries by screw fixation was troublesome. Besides, the volume of the distal tibiofibular space increased with the suture-button fixation technique should be taken into consideration.

Thornes et al. [18] published a retrospective cohort study including 16 patients treated with an early version of suture-button implant and 16 patients treated with traditional screw fixation. The fractures were classified as Weber-C in all cases. The patients in the suture-button fixation group showed significantly better AOFAS scores at 3 months (*p* = 0.01) and at 12 months (*p* = 0.04) postoperatively and earlier return to work than the screw fixation group (2.8 months versus 4.6 months, *p* = 0.02). In addition, most of the patients were satisfied with the suture-button device while a greater number of fair or poor results existed in patients who had syndesmosis screw fixation. They concluded that the suture-button device could accelerate rehabilitation and improve outcomes.

Cottom et al. [12] conducted a prospective cohort study, which consisted of 50 patients; 25 in the screw fixation group and 25 in the suture-button group. No statistically significant differences were identified in regard to time to postoperative weight bearing and subjective outcome scores between the fixation groups. Statistically significant improvements were noted in the modified AOFAS scores for each group between the preoperative and postoperative measurements (*p* < 0.05). Seventeen patients in the screw fixation group had a second operation for implant removal at an average of 4.38 months.

Coetzee et al. [15] published an ongoing randomized controlled trial, comparing syndesmosis screw fixation group with the suture-button fixation group. Each group consisted of 12 patients. It was unclear which fracture types were included. No significant differences were found regarding the AOFAS scores at a median 2.3-year follow-up. One patient in the suture-button group required removal at 6 months due to ongoing irritation and superficial infection, and 1 large fragment screw was removed due to the prominence of the screw head. The patients in the tightrope group have demonstrated non-significant better ROM measurements (*p* = 0.054) and subjectively reported less stiffness and discomfort.

Kim et al. [2] performed a comparison between TightRope fixation (*n* = 24) and screw fixation (*n* = 20). The fracture types were SER IV,SER III,SA I,SA II,PER IV,PER III,PA III, PA II. Regarding tibiofibular clear space, the improvement in the screw fixation group was statistically significant (*p* = 0.01),but the improvement in the suture-button fixation group was not (*p* = 0.05). However, the suture-button fixation group fully recovered to a normal ROM. Metal screws had broken in 5 of 24 patients (20.8%). No statistically significant difference was found between the two techniques according to AOFAS scores and VAS scores.

Seyhan et al. [17] performed a retrospective comparative study, including 7 Weber B fractures and 25 Weber C fractures with syndesmosis injury, of which 15 patients were treated with the TightRope system and 17 patients with the cortical screw system. No statistically significant difference was found between the two techniques according to AOFAS scores at the 3rd, 6th and 12th months. All cortical screws were routinely removed at the end of the 3rd month after operation. Six patients had TightRope device removal due to discomfort and soft tissue irritation. The TightRope fixation showed significant better results for the range of motion compared to screw fixation (*p* < 0.01).

### Functional evaluation

Several different scoring systems were applied to evaluate the functional outcome between the suture-button fixation group and the screw fixation group, such as: AOFAS score, the Olerud–Molander score, modified AOFAS scoring scale, the SF-12 health questionnaire, the Foot and Ankle Disability Index score, and so on. As the AOFAS score was the most used outcome, thus, we selected it to make a comparison. The AOFAS score of 150 patients treated with the suture-button device was 91.06 points, with an average follow-up of 17.58 months. The AOFAS score of 150 patients treated with syndesmotic screws was 87.78 points, with an average study follow-up of 17.73 months.

### Implant removal

The outcome of implant removal was reported in 7 studies [4, 8, 9, 12, 15, 17, 18]. We excluded one study which made a routine screw removal [17]. Implant removal was reported in 5 of 134 (3.7%) patients treated with the suture-button device, and in 54 of 134 (40.2%) patients treated with the syndesmotic screw.

### Implant failure

Seven publications demonstrated implant failure [2, 4, 8, 9, 12, 15, 17]. No patient in the suture-button fixation group had implant failure, however 48 of 155 (30.9%) patients treated with the syndesmotic screw suffered from implant failure.

### Malreduction

The outcome of malreduction was reported in 4 studies [8, 9, 11, 17]. Malreduction was reported in 1 of 93 (1.0%) patients treated with the suture-button device, and in 12 of 95 (12.6%) patients treated with the syndesmotic screw.

### Post-operative complications (except implant failure and malreduction)

Though the implant failure and malreduction are considered as post-operative complications, we have already interpreted them respectively. On current section, we preferred to make a comparison of other complications such as infection, soft-tissue irritation, discomfort, syndesmosis ossification and so on. The outcome of complications was reported in five studies [4, 8, 9, 15, 17]. 13 of 108 patients (12.0%) treated with the suture-button device were reported with post-operative complications and 18 of 110 patients (16.4%) in the screw fixation group.

### Potential cost and cost effectiveness

In our search, only one publication [16] was identified which made a cost-effectiveness analysis between suture button and syndesmotic screws fixation for unstable SER IV ankle fractures. This publication demonstrated that suture button fixation was more cost-effective than syndesmotic screws without a routine removal. Patients treated with the suture button device spent on average $1482 less and had a higher quality of life by 0.058 quality-adjusted life-year compared with patients who treated with two syndesmotic screws. Assuming that functional outcomes and failure rates were equivalent, the screw fixation only became more cost-effective when the screw hardware removal rate was reduced to less than 10% or when the suture button cost exceeded $2000. In addition, fixation with a single suture button device proved more cost-effective than fixation with either 1 or 2 syndesmotic screws.

## Discussion

Though current gold standard to treat syndesmotic injuries is syndesmotic screw fixation, using a suture-button technique has been raised more and more interest and increased rapidly over the last decade. In the current review suture-button fixation group shows similar AOFAS outcome scores (91.06 points) compared to conventional screw fixation (87.78 points) group. The rate of implant removal and malreduction was lower in the suture-button fixation group. Besides, no patient in the suture-button fixation group had implant failure, however the rate of implant failure in the screw fixation group was 30.9%. In addition, the rate of post-operative complications was lower in the suture-button fixation group (12.0% versus 16.4%). There was only one publication demonstrated about cost-effectiveness aspects, it concluded that using a suture-button device is more economical than syndesmotic screws not removed on a routine basis in the treatment of supination-external rotation type 4 injuries.

The need for routine syndesmotic screw removal and the time to screws removal are still controversial. All publication except one [15] in this current review demonstrated a lower implant removal rate in the suture-button group. Our result was on the basis of no routine screw removal. A second operation for implant removal could lead to potential infections, an increased cost to the patient, missed work days, or other complications [19, 20]. Routine removal of the syndesmosis screw(s) has been reported with additional cost for a second procedure and for the treatment of potential complications [20, 21]. Besides, literature has demonstrated that early screw removal before ligamentous healing is accompanied with risk of developing recurrent syndesmotic diastasis [22]. In a review, wound infection was observed in 9.2% of the cases and recurrent syndesmotic diastasis in 6.6% after removal of syndesmosis screws [22]. Schepers T, et al. demonstrated that there was no favorable outcome when electively removing syndesmosis screws [23]. According to the publication of Kortekangas et al. [8], syndesmotic screw was broken in three patients and intact but loosened in 13 patients at the final follow-up, but just 3 of them resulted in malreduction. Though local symptoms may be developed if the screw is not removed and remains unbroken [24], routine removal of the syndesmosis screw or not is still debatable, which indicates the need for additional high-quality studies comparing routine removal and removal on indication. The suture-button technique is theoretically accompanied with no need of implant removal. However, the removal of the suture-button device was described in several studies with different percentages ranging from 0% to 13.3% in current review, and 3.7% on average. The main reason of implant removal was implant irritation. Naqvi et al. [25] demonstrated that after a slight modification (embedding of the knot at the lateral side) of the surgical technique in 31 of the 49 patients, causes no removal of the TightRope device.

Many previous investigations evaluating TightRope fixation for syndesmotic injuries have reported 0% malreduction rates, but they used only plain radiography to assess malreduction [9, 25–28]. Naqvi et al. [11] compared syndesmotic screw and TightRope fixation using CT of both ankles to assess syndesmotic reduction and found no malreduction in the TightRope fixation group with a mean follow-up time of 2.5 years. The suture-button technique is theoretically accompanied with no need of implant removal; thus, recurrent syndesmotic diastasis is less likely to occur. Even when the suture-button device required removal, no loss of reduction of the syndesmosis was observed [9, 17]. Anand et al. through a multicenter case series consisted of 36 patients demonstrated that the ankle tightrope maintained satisfactory reduction in the ankle mortise in 97% of cases with a mean follow-up of 14 months [29].

The main complications reported in the included studies were infection, soft-tissue irritation, discomfort, syndesmosis ossification and so on. In current review, the suture-button fixation group showed similar risk of post-operative complications with the screw fixation group. Regarding the suture-button fixation group, some literatures suggested some modifications in surgical procedure, such as a posterior short knot and/or reaming the posterior aspect of fibula which was useful to reduce the incidence rate of infection, irritation and discomfort [25, 30]. These complications existed as similar issues to the screw fixation group. Laflamme et al. [9] reported that one patient in each group demonstrated partial syndesmosis ossification without complete synostosis with a follow-up of 12 months. DeGroot et al. [31] demonstrated that the application of suture-button device accompanied with complications such as osteolysis, enlargement of the tibia drill-hole and subsidence of the device with an average follow-up of 20 months. Fantry et al. described 3 patients with TightRope fixation for syndesmotic instability who developed deep infection, they considered that braided suture within suture button devices could provide an environment advantage to the development of infection across the syndesmotic fixation tract and the evidence of suture button migration or osteolysis of the TightRope tract prompt an infectious workup and need of implant removal. When there is a concern for infection, it is essential to remove both metallic buttons and the entirety of the suture to prevent further infection [32].

The dynamic nature of the suture button device theoretically could allow some degree of physiologic micromovement of the syndesmosis, leading to earlier return to full weight-bearing and better objective ROM measurements. However, screw fixation does not allow normal motion of the syndesmosis during healing because the screw may break or loose. Thornes et al. noted that patients in the suture-button group were kept no weightbearing for a significantly shorter mean time than patients in the syndesmotic screw group (4.1 weeks versus 6.3 weeks, *p* = 0.01) with no patients in the suture-button group required implant removal [18]. Naqvi et al. found that the TightRope group needed a shorter time to full weightbearing with no case of malreduction was observed (8.0 weeks versus 9.1 weeks). Cottom et al. also showed a shorter mean time to full weight-bearing in the TightRope group with no cases of implant failure and implant removal. Degroot et al. [31] reported an average time to full weight-bearing of 5.7 weeks using TightRope, with no signs of implant failure or residual displacement at a follow-up of 20 months. Cottom et al. and Thornes et al. both demonstrated that fast full weight-bearing could bring an accelerated rehabilitation [12, 18]. Some literatures showed the patients in the tightrope group had better objective range of motion (ROM) measurements [9, 15]. Laflamme et al. [9] demonstrated that the ankle range of motion was higher in the dynamic fixation group at all times when considering the plantar flexion, although this difference was minor for dorsal flexion [9]. Interestingly, some included studies reported that patients in the dynamic fixation group seemed to present with less pain and discomfort which maybe contributed to earlier full weight-bearing [9, 15].

Regarding the cost-effectiveness aspect, the following items should be taken into consideration: second surgery for implant removal, potential complications, number of follow-up clinic appointments, and time to return to work. Many studies have already shown lower risk of implant removal and implant failure by using the suture-button device, which theoretically means less medical costs. Besides, some literatures have described that patients in the suture-button group returned earlier to their previous working level [9, 18]. Literature has reported that the additional costs of a syndesmotic screw removed in daycare surgery in the Netherlands are around 700 Euro, which is approximately the cost of two TightRope systems [3]. Besides, it was clearly shown in the Toowoomba Hospital Australia, that by using the TightRope system instead of conventional syndesmosis screws there is a cost saving of $651.50 AUD per case which was based on a second operation for screw removal [33]. However, there is currently no prospective research on the cost-effectiveness of the TightRope device versus a syndesmotic screw.

### The key aspects for future research

1). As function outcomes are influenced by severity of trauma, presence of cartilage injury, soft tissue healing, subjective sensation of patients and so on, it is more appropriate to assess syndesmotic injuries based on radiologic parameters rather than functional scores. The key point is the accurate anatomic reduction of the syndesmotic injuries. Thus, more high-quality studies comparing the reduction outcomes of screw fixation and suture-button fixation should focus on radiologic evaluation. 2). Computed tomography as a useful tool for the detection of minor syndesmotic diastasis should be mostly used to make the assessment of postoperative malreduction [4, 34]. Bilateral CT investigations are suggested to act due to anatomic variations [8, 35, 36]. Besides, 3-dimensional volume investigation of syndesmotic space might be more appropriate than measuring the distal tibiofibular space at a single level [37]. 3). Complications like deep infection, osteolysis, subsidence of the implant and enlargement of the tibial drill-hole in the usage of suture-button device, have been noted at longer follow-up. Thus, studies concerning suture-button fixation should focus on effectiveness and safety at long-term follow-up. 4). Whether using one or two suture-buttons, the shape of button, and the placement of suture-buttons are need to make a further research. It was recommended to use 2 TightRope devices when treating Maisonneuve fractures [38]. 5). As the sample size of most published studies was small, thus, larger prospective controlled studies are required to furtherly prove the advantages of the suture-button fixation to ankle joint kinematics and function. 6). To prove superiority of the TightRope system furtherly, it should be compared in randomized controlled trials with syndesmotic screws removed only on clinical indications. 7). Prospective studies on the hospital and socioeconomic cost-effectiveness of the TightRope system versus a syndesmotic screw are desirable.

### Limitations identified with this study

1). The publication of Coetzee et al. [15] was an ongoing randomized controlled trial with incomplete outcome, besides, the methods of random sequence generation, the adequate concealment of allocation, double- blinding, the blinded assessments of the results were unclear, which could make a significantly influence on the stability of the outcomes; 2). The differences in study types, fracture types, surgical technique, postoperative measures and the uniformity in outcome reporting decreased the credibility and the stability of the outcomes; 3). The sample size of included studies was relatively small, especially the publication of Coetzee et al.; 4). The publication bias exists.

## Conclusions

Based on our research, though the suture-button fixation group had similar functional outcomes (measured on the AOFAS score) and post-operative complication rate compared with the syndesmotic screw fixation group, the suture-button device could lead to better objective range of motion measurements and earlier return to work. Besides, the suture-button fixation group had lower rate of implant removal, implant failure, and malreduction. However, high-quality randomized controlled trials with more uniformity in outcome reporting are desirable to determine the long-term effects and cost-effectiveness of the suture-button device.

## Abbreviations

AOFAS: American Orthopaedic Foot and Ankle Society ankle score
FADI: Foot and Ankle Disability Index
RCTs: Randomized controlled trials
ROM: Range of motion
VAS: Visual Analogue Scale
